# Reshaping maternal services in Nigeria: any need for spiritual care?

**DOI:** 10.1186/1471-2393-14-196

**Published:** 2014-06-06

**Authors:** Abiodun I Adanikin, Uche Onwudiegwu, Akinyemi A Akintayo

**Affiliations:** 1 Department of Obstetrics and Gynaecology, Ekiti State University, Ado-Ekiti, Nigeria; 2 Department of Obstetrics, Gynaecology and Perinatology, Obafemi Awolowo University, Ile-Ife, Nigeria; 3 P.O. Box 1799 Lagere, Ile-Ife, Nigeria

**Keywords:** Pregnancy, Childbirth, Hospital, Spiritual care

## Abstract

**Background:**

High maternal and perinatal mortalities occur from deliveries conducted in prayer houses in Nigeria. Although some regulatory efforts have been deployed to tackle this problem, less attention has been placed on the possible motivation for seeking prayer house intervention which could be hinged on the spiritual belief of patients about pregnancy and childbirth. This study therefore seeks to determine the perception of booked antenatal patients on spiritual care during pregnancy and their desire for such within hospital setting.

**Method:**

A total of 397 antenatal attendees from two tertiary health institutions in southwest Nigeria were sampled. A pretested questionnaire was used to obtain information on socio-demographic features of respondents, perception of spiritual care during pregnancy and childbirth; and how they desire that their spiritual needs are addressed. Responses were subsequently collated and analyzed.

**Results:**

Most of the women, 301 (75.8%), believe there is a need for spiritual help during pregnancy and childbirth. About half (48.5%) were currently seeking for help in prayer/mission houses while another 8.6% still intended to. Overwhelmingly, 281 (70.8%) felt it was needful for health professionals to consider their spiritual needs. Most respondents, 257 (64.7%), desired that their clergy is allowed to pray with them while in labour and sees such collaboration as incentive that will improve hospital patronage. There was association between high family income and desire for collaboration of healthcare providers with one’s clergy (OR 1.82; CI 1.03-3.21; p = 0.04).

**Conclusion:**

Our women desire spiritual care during pregnancy and childbirth. Its incorporation into maternal health services will improve hospital delivery rates.

## Background

Even though historically women in labour have been supported by relatives or other women, contrary is the worldwide practice in our hospitals today. Continuous support during labour has become the exception rather than the routine [1]. Gradual dehumanization of the maternity services has occurred overtime with little attention paid to patients’ psychosocial supports of which spiritual care is integral.

There has been increasing interest in the relationship between spirituality and clinical care in the last two decades [2,3]. This is because it is believed that a whole person has physical, emotional, social and spiritual dimensions. To neglect any of these therefore may negate a critical part of patient-centred medicine which is the backbone of high quality healthcare [4,5].

Spirituality has been defined as the ways in which a person habitually conducts his or her life in relationship to the question of transcendence [6]. Evidence has shown that this could hugely impact on the ability of a patient to cope with medical conditions, provide a source of comfort and supply a font of wisdom in difficult situations [7]. Notwithstanding, there is no consensus on the role medics can play in attending to patients’ spiritual needs [4,6,8].

In developing countries, significant proportion of births takes place in mission homes, prayer houses, churches and mosques. Publications from Nigeria have highlighted that activity of some prayer houses significantly contribute to unsafe motherhood [9,10]. Maternal mortality ratio (MMR) of 978/100,000 births and perinatal mortality rate (PNMR) of 118/1000 total birth have been quoted to emerge from these places. This is because deliveries are often undertaken by unskilled attendants and in unhygienic environments [11].

Although there is low patronage of hospital antenatal services in the country [12], a chunk of booked patients still end up delivering in prayer houses. There, labour complications are commonly blamed on the parturient, accusing her of witchcraft, unfaithfulness to her husband, etc [11].

Till date, most researchers in our environment have focused primarily on illiteracy, financial constraint, fear of possible caesarean section, industrial action by health workers, transport difficulty at night and communal disputes as reasons for patronizing prayer homes [11,13]; however little attention has been paid to the spiritual belief/perception of patients about pregnancy and childbirth as the basis for seeking prayer house intervention.

By keeping a patient’s spiritual need and belief separate from her care, we may be ignoring a core aspect of support system [4]. This study therefore aims to determine from the booked antenatal patients their perception of spiritual care during pregnancy and childbirth and how they desire it is addressed. Knowledge of this might engender policy on maternal services that will enhance hospital patronage and delivery among women.

## Methods

This multicentre cross-sectional study was conducted between 1^st^ of February and 31^st^ of May, 2013 at the Obstetrics and Gynaecology unit of Bowen University Teaching Hospital (BUTH), Ogbomoso and Ekiti State University Teaching Hospital (EKSUTH), Ado-Ekiti, southwest Nigeria.

BUTH is a privately owned tertiary health institution located in Oyo State while EKSUTH is a government-owned tertiary health facility in Ekiti State. Both centres receive referrals from Oyo, Osun, Ondo and Ekiti zones of south-west Nigeria.

EKSUTH runs antenatal clinic twice a week and has average birth rate of 2000 per year. On the other hand, BUTH holds antenatal clinic once in a week, average delivery rate hovers around 1,000. Both institutions do not routinely provide spiritual services with their maternity care. Ethical clearance for the study was obtained from the ethics committee of EKSUTH (A67/2013/0801).

All booked antenatal patients at the hospitals during the study period were eligible for recruitment. Informed written consent was obtained from participants. Out of 446 eligible pregnant women approached from both institutions, a total of 397 women agreed to participate in the study (overall response rate was 89%). BUTH had a response rate of 86% (131 out of 153 approached) while EKSUTH had response rate of 91% (266 out of 293 approached).

A self-administered questionnaire developed from the HOPE Questions practical tool guide [14] was used to obtain information from respondents. The HOPE questions guide was not previously validated but the strength of this approach is that it allows for an open-ended exploration of an individual’s spiritual concerns and serve as a natural follow up to isolate other support systems. The developed questionnaire was however pretested at the antenatal clinics of both EKSUTH and BUTH prior to the commencement of study in order to remove ambiguity. Content validity was ensure by translating the questionnaire to major local languages and back translated into English.

The questionnaire elicited information on socio-demographic variables of respondents, perception of spiritual care during pregnancy and childbirth, personal spiritual practices, role of organized religious denominations in pregnancy care; and how the patients desire that their spiritual needs are addressed. A trained resident doctor was available to assist respondents who had difficulty in answering any of the questions.

Collected data were collated for subsequent analysis using statistical package for social sciences (SPSS) version 15 (IBM, Armonk, NY, USA). Frequency tables were generated and logistic regressions were used to test for association between socio-demographic variables of respondents and their desire for health provider’s collaboration with personal clergy. The level of significance (α) was set at 0.05.

## Results

Table 1 shows the socio-demographic features of the respondents. Their age ranged from 16 to 45 years with a mean of 29.67 ± 4.87 years. Two hundred and seven (52.1%) were Christians while 190 (47.9%) were Muslims. Most respondents, 235 (59.2%), had family monthly income that is less than $500; just a fifth (20.2%) earn above $1000.

**Table 1:** Socio-demographic features of respondents

**Characteristics**	**n (%)**	**Mean**	**Range**
**Age (years)**		29.67 ± 4.87	16-45
15-24	51 (12.9)		
25-34	284(71.5)		
≥35	62(15.6)		
			
**Occupation**			
Unskilled	150(37.8)		
Semi-skilled	91(22.9)		
Skilled	156(39.3)		
			
**Marital status**			
Single	28(7.0)		
Married	368(92.7)		
Widowed	1(0.3)		
			
**Religion**			
Christianity	207(52.1)		
Islam	190(47.9)		
			
**Tribe**			
Yoruba	330(83.1)		
Igbo	53(13.3)		
Hausa	14(3.6)		
			
**Educational level**			
None	17(4.3)		
Primary	42(10.6)		
Secondary	93(23.4)		
Tertiary	245(61.7)		
			
**Husband’s educational status**			
None	8(2.0)		
Primary	19(4.8)		
Secondary	47(11.8)		
Tertiary	323(81.4)		
			
**Family monthly income**			
< $100	131(33.0)		
$100-500	104(26.2)		
$501-1000	82(20.6)		
> $1000	80(20.2)		
			

Perception of respondents to spiritual care in pregnancy and childbirth is shown in Table 2. Majority of the patients, 286 (72.0%), belonged to churches or mosques that provide spiritual support during pregnancy and/or childbirth. Mainly, the support was prayers alone. However, 41 (10.3%) are in denominations that provide midwifery services while another 55 (14.1%) provide a combination of prayers and midwifery services. A sizable proportion, 301 (75.8%), of respondents believe there is a need for spiritual help during pregnancy and childbirth. Close to half of the patients (48.5%) were currently seeking such help while another 8.6% intended to.

**Table 2:** Respondents’ perception of spiritual care

**Issues**	**Response [n (%)]**
**Does your denomination provide spiritual support during pregnancy/child birth?**	
Yes	286(72.0)
No	111(28.0)
**If yes, which spiritual support?**	
Prayers	190(47.9)
Midwifery services	41(10.3)
Both	55(14.1)
	
**Do you believe there is need for spiritual help during pregnancy/ childbirth?**	
Yes	301(75.8)
No	96(24.2)
**Are currently seeking spiritual help?**	
Yes	193(48.5)
No	170(42.8)
No, but intends to.	34(8.6)
	
**Do you think doctors/nurses should consider patient’s spiritual needs/concerns during medical care?**	
Yes	281(70.8)
No	116(29.2)
**How often have clinicians discussed spiritual help with you?**	
Frequently	24(6.0)
Occasionally	167(42.1)
Never	206(51.6)
	
**Do you desire that healthcare providers allow your clergy to pray with you while in labour?**	
Yes	257(64.7)
No	109(27.5)
Indifferent	31(7.8)
**Do you think collaboration of healthcare providers with your spiritual leader/clergy will improve your chance of hospital delivery?**	
Yes	247(62.2)
No	85(21.4)
Not sure	65(16.4)
	
**Are their spiritual practices you desire that hospitals allow during labour?**	
Prayer	257(64.7)
Listening/reading scriptures	47(11.9)
Listening to religious music	37(9.3)
Others	29(7.3)
None	27(6.8)

Although 116 (29.2%) of the women felt there was no need for doctors/nurses to consider their spiritual needs during medical care, 281 (70.8%) felt it was needful. Majority, 257 (64.7%) desired that their clergy be allowed to pray with them while in labour. The survey further showed that 247 (62.2%) of the respondents think that collaboration of healthcare providers with their clergy will improve their chances of delivering in the hospital. Virtually all participants had a spiritual practice that they wish the hospital will allow.

For example, a respondent stated:

‘*My last delivery was a difficult moment for me. At a point I lost hope in having a successful outcome especially when the nurses were saying something about the fetal heart dropping. Chanting the scriptures to my hearing can build my faith and allay my fear. I desire that this is allowed in my next labour’.* One participant wrote, *‘…..I will be glad if my pastor is allowed to anoint me and pray for me’.* Another woman indicated, *‘We have a church prayer band, if they can have a place within the hospital to intercede while I’m in labour that will be nice’.*

Following logistic regression analysis, those with unskilled job and monthly income > $1000 had increased desire for health provider’s collaboration with personal clergy (Table 3).

**Table 3:** Association between socio-demographic features of respondents and desire for health-provider collaboration with own clergy

**Variable**	**Desires collaboration with clergy**		**Adjusted**		
	**Yes**	**No/Not sure**	**Odd ratio**	**95% CI**	**P value**
**Age (years)**					
15-24	35(68.6)	16(31.4)	1.44	0.78-2.78	0.25
25-34	170(59.9)	114(40.1)	1.00		
≥35	42(67.7)	20(32.3)	1.39	0.78-2.52	0.24
**Occupation**					
Unskilled	84(56.0)	66(44.0)	1.61	1.02-2.57	0.04
Semi-skilled	58(63.7)	33(36.3)	1.17	0.68-2.02	0.57
Skilled	105(67.3)	51(32.7)	1.00		
**Marital status**					
Single	17(60.7)	11(39.3)	1.08	0.49-2.34	0.87
Married	229(62.2)	139(37.8)	1.00		
Widowed	1(100)	0(0)	0.55	0.02-13.56	0.71
					
**Religion**					
Christianity	124(59.9)	83(40.1)	1.00		
Islam	123(64.7)	67(35.3)	0.81	0.54-1.22	0.32
					
**Tribe**					
Yoruba	207(62.7)	123(37.3)	1.00		
Igbo	33(62.3)	20(37.7)	1.03	0.56-1.86	0.95
Hausa	7(50.0)	7(50.0)	1.68	0.57-4.91	0.34
					
**Educational level**					
None	10(58.8)	7(41.2)	1.32	0.48-3.42	0.61
Primary	28(66.7)	14(33.3)	0.94	0.46-1.85	0.82
Secondary	50(53.8)	43(46.2)	1.59	0.98-2.58	0.06
Tertiary	159(64.9)	86(35.1)	1.00		
**Husband educational status**					
None	5(62.5)	3(37.5)	1.07	0.24-4.32	0.98
Primary	12(63.2)	7(36.8)	1.01	0.38-2.57	0.98
Secondary	27(57.5)	20(42.5)	1.26	0.67-2.33	0.48
Tertiary	203(62.8)	120(37.2)	1.00		
					
**Monthly income**					
< $100	86(65.6)	45(34.4)	1.00		
$100-500	65(62.5)	39(37.5)	1.15	0.67-1.96	0.62
$501-1000	55(67.1)	27(32.9)	0.94	0.52-1.68	0.83
> $1000	41(51.3)	39(48.7)	1.81	1.03-3.21	0.04

## Discussion

Our study found that most participants believed there is a need for spiritual care during pregnancy/childbirth. Close to half of them were already seeking such care in prayer/mission houses while receiving hospital care. Two-thirds felt healthcare providers should consider their spiritual needs during medical care, though that has been neglected till date.

This finding supports conclusion of previous surveys that suggested a need to incorporate the spiritual and religious dimension of patients into clinical managements [15,16]. Though prevalence varied with settings, evidence from past studies has indicated that 33% to 77% of patients expect clinicians to attend to their spiritual needs [6]. *Tanyi*[17] actually submitted that one cannot truly assess a childbearing woman without assessing her spirituality. However, the observation is at variance to assertions of some critics that religion is either irrelevant or sometimes even harmful to medical progress and good clinical care [18,19]. Despite the contradiction, a careful consideration of patient’s background and values is still important in the course of clinical care.

In Africa for example, some women view pregnancy as a vulnerable time when there is increased risk of being affected by witchcraft. This mentality makes the women seek protection and, a prayer house is sometimes seen as safe haven [13,20]. Women from developing countries also believe in the significance of higher power in influencing birth outcomes [21]. To optimally manage such patients and gain their confidence, it will be essential to consider their beliefs, expectations and preferences.

In this study participants expressed a strong desire for a health system that has a provision for personal clergy to be able to pray while in labour and collaboration with one’s clergy was seen as incentive that will encourage hospital delivery.

Different religions have practices that are held sacred during labour and childbirth. In Islam, spiritual practices such as using of zam-zam water, reading of the Qu’ran and whispering a prayer, Adhan, in the right ear of the infant immediately after birth to protect against ‘evil eye’ are highly revered [22]. Christians place premium on profession of biblical promises, prayers, use of anointing oil, mantles, etc in the course of delivery. A faith-tolerant hospital environment therefore might encourage more women to patronize orthodox healthcare, since allowance for prayers and other spiritual practices will be concomitantly available. It may be a key to improving utilization of maternal services in Nigeria and the sub-Saharan Africa as a whole.

Learning to handle spiritual aspects of medical care is not a typical part of medical school training. More so, wide variety of spiritual backgrounds of patients will raise important practical questions of how the spiritual needs will be met. Suggested best way is by trained clergy [4,8]. It will be good to adopt the policy of having hospital chaplains in our environment. But more importantly, patient’s personal clergy may be in the best position to provide the needed support based on peculiarity of different faith communities. Clinicians should be able to elicit spiritual history and make appropriate collaborative efforts or referrals. We can actually work with the clergies in non-competitive ways to benefit our patients [23].

The question of moral obligation of health providers in attending to spiritual concerns of patients and ethical boundaries of such intervention readily comes to mind. Since health professionals have sworn to treat patients to the best of their ability and judgment, holistic management approach is still in conformity with the oath [24]. Ethically, while pursuing spiritual discussion, the safest rule is to follow patient’s lead. A balanced, open-minded approach must be maintained without sacrificing scientific integrity [6,14].

The study found association between high family income and desire for collaboration of healthcare providers with one’s clergy (OR 1.82; CI 1.03-3.21; p = 0.04). This shows that being financially buoyant does not eliminate the entrenched belief in the intervention of deity.

This study is not without limitation. The choice of study sites, proportionate allocation of study population and recruitments of participants was by convenient sampling, however, this does not invalidates the research findings. For future purpose, a more detailed research process will be suggested.

## Conclusion

Our current maternal services pay little or no attention to patients’ psychosocial support despite evidences from Cochrane review that it is beneficial [1]. From this study, it is clear that patients desire that spiritual care is offered alongside with medical treatments. The approach could be an attracting force to improve hospital maternity patronage.

In order to ensure safer motherhood and reduce prevailing high morbidity and mortality rate in a third world country like ours, hospital delivery must improve. It is therefore recommended that our current maternal services be restructured to make provision for spiritual care since this would not interfere with the usual core scientific clinical care provided to the women.

## Abbreviations

BUTH: Bowen University Teaching Hospital; EKSUTH: Ekiti State University Teaching Hospital.

## Competing interests

This research has been self-funded and we declare that we have no conflict of interest.

## Authors' contributions

AIA was responsible for study concept, design and manuscript writing. UO and AAA critically reviewed the manuscript for intellectual content and gave final approval of the version to be published. All authors read and approved the final manuscript.

## Pre-publication history

The pre-publication history for this paper can be accessed here:

http://www.biomedcentral.com/1471-2393/14/196/prepub
